# Clinician Perspectives on Incorporating Physical Activity and Sleep Prescriptions Using eHealth for Youth With Comorbid Psychiatric Disorders: Qualitative Focus Group Study

**DOI:** 10.2196/71569

**Published:** 2025-11-07

**Authors:** Alyssa M Button, James Slavet, Amanda E Staiano, April B Bowling

**Affiliations:** 1 Virginia Commonwealth University Richmond, VA United States; 2 Pennington Biomedical Research Center Baton Rouge, LA United States; 3 Marblehead Family Therapy and Wellness Marblehead, MA United States; 4 Merrimack College North Andover, MA United States

**Keywords:** mental health, health coaching, mobile health, mHealth, attention-deficit/hyperactivity disorder, ADHD, depression, autism

## Abstract

**Background:**

Physical activity and sleep prescriptions are indicated for the treatment of psychiatric disorders among youth. However, there is limited clinical adoption of these practices. Exergaming (ie, games that require physical activity) is a feasible intervention to promote physical activity and sleep hygiene and is appealing to youth given their interest in video gaming. Integrating exergaming prescriptions into clinical mental health practices may offer an opportunity to expand access to these interventions, yet pragmatic considerations for adopting these programs are poorly understood.

**Objective:**

This study aimed to gain feedback from practicing clinicians on adopting GamerFit, an app-based intervention that incorporates exergames, step and sleep tracking, and online coaching to promote physical activity and sleep, as a tool in treatment plans for youth aged 13 to 17 years with psychiatric disorders.

**Methods:**

Mental health clinicians participated in 2 online focus groups. A semistructured interview collected information on perceptions of the importance of physical activity and sleep, considerations for using GamerFit with clients, and approaches for incorporating GamerFit into standard care. Qualitative analysis included a hierarchical thematic coding system of isolated quotes, with the structure, frequency, and interrelationships of the coded quotes used for analysis.

**Results:**

All clinicians (8/8, 100%) endorsed physical activity and sleep prescriptions as important interventions, although they were not typically a focus of treatment. Clinicians reported varying levels of self-efficacy in encouraging physical activity goals (6/8, 75%) and, to a lesser extent, sleep hygiene (4/8, 50%). Most perceived eHealth approaches positively (7/8, 88%) and noted their appeal given the accessibility of this physical activity option via gaming (2/4, 50%). Clinicians were optimistic about the feasibility of using GamerFit; the exergame and health coaching aspects of GamerFit were perceived favorably (5/8, 62%). Clinicians desired to access app data in electronic health systems to incorporate in therapeutic sessions (4/8, 50%) and recommended using the app in residential settings with continued use at home (2/8, 25%). Clinicians expressed concern regarding the implementation of GamerFit with families with low technology literacy, noting that some patients would likely require parental assistance to help with reminders and technology use (1/8, 12%). Suggestions for improvement included a greater variety of exergames and features to increase adolescents’ engagement (6/8, 75%). There was a considerable willingness to incorporate this technology into clinicians’ clinical practices and a strong desire for insurance provisions to cover coaching and technological components (7/8, 88%).

**Conclusions:**

Clinicians perceived GamerFit as a feasible and acceptable clinical approach to physical activity and sleep prescriptions for youth with psychiatric disorders. The remote delivery of this intervention was perceived to be of interest to patients and provided helpful guidance for clinicians who were short on time to address many important topics within limited session time frames.

## Introduction

Physical activity in adolescence is well known to promote optimal physical and mental health outcomes both immediately and over the long term [1-5]. Despite the benefits, most youth in the United States do not meet recommendations set forth by the *Physical Activity Guidelines for Americans, 2nd Edition* [6,7]. Youth with psychiatric disorders, who represent 1 in 5 children and adolescents in the United States per year [8], fall even further behind [9], contributing to a disproportionate risk of chronic disease [10]. Importantly, physical activity and sleep are bidirectionally associated, where physical activity contributes to improved sleep quality and duration, and vice versa [11]. Sleep disturbance rates are notably higher among youth with psychiatric conditions and likely exacerbate mental health symptoms [12], highlighting the value of treatments that can address both low physical activity and poor sleep simultaneously.

Youth with psychiatric disorders face unique barriers that reduce the likelihood of participating in physical activity programming, such as social isolation and peer exclusion, dislike of competition, preference for solitary activity, motor delays, dysregulation, medication side effects, and negative experiences with previous programming [13]. For youth with physical, cognitive, or self-regulatory challenges, additional parent or caregiver support and action may be required for engagement in physical activity [14]. There is a critical need for health programming to reduce chronic disease disparities in this population through the adaptation and use of behavioral health programming that overcomes these reported barriers by providing pragmatic support to parents and youth in multiple settings [14].

Physical activity interventions, in conjunction with traditional mental health treatment (ie, psychotherapy), have been shown to improve symptoms related to psychiatric conditions such as autism spectrum disorder [15], depression [16], anxiety [17], and attention-deficit/hyperactivity disorder [18]. Improvements in sleep similarly benefit a wide range of cognitive, affective, and behavioral outcomes in youth with psychiatric disorders [19]. For these reasons, mental health treatment providers are encouraged to use exercise as medicine through behavioral prescriptions [20] and to promote positive sleep hygiene [21]. Despite evidence of the importance of physical activity and sleep in mental health treatment, there are mixed reports of clinician uptake, with several studies documenting that physical activity interventions remain underused [22] and only a small proportion of clinicians providing recommendations that are consistent with scientific evidence and national guidance [23]. This may be due, in part, to a lack of clinician training and practical barriers for clients [24].

GamerFit is a theory-based exergaming (ie, gaming that requires physical exertion) and telehealth physical activity coaching intervention that has shown efficacy in promoting physical activity and sleep [25,26] and consequently in producing positive psychosocial outcomes (ie, self-regulation, affect, health related quality of life, and social support) among youth aged 13 to 17 years with psychiatric diagnoses [27]. It was adapted from the evidence-based GameSquad intervention designed for youth with overweight and obesity [25]. Adaptations were made to accommodate the needs of youth with heterogeneous and comorbid psychiatric diagnoses based on disability-informed empowerment theory [28]. This 12-week intervention uses a mobile app that incorporates telehealth coaching, exergaming challenges, and on-demand exercise videos to increase physical activity levels and improve sleep hygiene [25]. Exergames are video games that require exertion or physical activity from the user and may be more pleasant and enjoyable than traditional modes of physical activity based on user characteristics, including leisure time preferences [29]. GamerFit participants use an app that includes a menu of exergames to select (and then play the games on a separate video game console); progressive physical activity and sleep hygiene planning and tracking; step tracking that automatically syncs with a Fitbit (Fitbit Inc), which automatically uploads data to the app for self-monitoring and coach monitoring; real-time automated feedback; prerecorded videos using peer modeling to promote health behaviors; and preset motivational texting encouraging youth to engage in at least 60 minutes per day of moderate-to-vigorous physical activity and to achieve 8 to 10 hours per day of quality sleep. App users, with an accompanying caregiver, schedule telehealth sessions with a trained coach once weekly for 12 weeks, to set physical activity and sleep goals and to overcome barriers to achieving weekly goals. The coaches are trained in motivational and goal-setting principles and, importantly, do not need to have a specific license or background in mental health treatment or exercise physiology. The app and intervention were designed by the research team and developed by a third-party developer; it is not available in the public domain. This intervention offers a solution to overcome youth and family barriers to engagement in physical activity by providing structured, child-focused programming in the home setting and accountability with a telehealth coach.

The next step of this work was to translate GamerFit for clinical use to improve accessibility for youth with psychiatric disorders. To do so, the purpose of this study was to explore clinician feedback on incorporating GamerFit into ongoing mental health treatment, ultimately to help clinicians make physical activity and sleep prescriptions.

## Methods

### Participants

Practicing mental health clinicians were recruited via email listservs and word-of-mouth referrals to participate in one of two 75-minute focus groups to gather perceptions of the GamerFit intervention as an adjunct to their ongoing mental health treatment practice. Participants were required to be currently practicing clinical pediatric mental health providers who were willing and able to participate in an English-language focus group. A convenience sample of participants was identified based on their clinical background and their response to recruitment attempts within the given 6-month time frame, and the remotely delivered groups were scheduled around the clinicians’ availability. After the second focus group, analysis indicated thematic saturation; therefore, no additional participants were recruited.

### Procedures

Focus groups were held via Zoom (Zoom Communications, Inc), a Health Insurance Portability and Accountability Act (HIPAA)–compliant web and videoconferencing platform, and were facilitated by trained study team members, including a clinical psychologist and a research scientist with experience delivering health promotion interventions for youth with psychiatric diagnoses. The interviewers were careful to avoid leading questions about the app and instead encouraged participants to share suggestions for improvement and practical considerations for implementation in clinical practice. Participants were presented with an overview of the GamerFit intervention components, which included a video demonstration of the user experience. Participants then asked questions about the GamerFit intervention. Next, a semistructured, empowerment theory-based instrument was administered with questions regarding (1) perceptions of using physical activity and sleep prescriptions in youth mental health treatment plans, (2) considerations for implementation of GamerFit in mental health treatment plans (ie, feasibility, acceptability, accessibility, and implementation fidelity), and (3) approaches for delivering physical activity and sleep prescriptions via the GamerFit intervention (Multimedia Appendix 1). These procedures did not change over the course of the study. The Standards for Reporting Qualitative Research guidelines [30] were followed in drafting and editing this manuscript (Multimedia Appendix 2).

### Ethical Considerations

All study procedures were approved by the Pennington Biomedical Research Center Institutional Review Board (2021-071-PBRC GamerFit). Participants provided verbal consent to participate and be recorded during the session. All data in this study were deidentified, and indirect identifiers were coded to prevent the possibility of reidentification. Participants were provided with a US $50 gift card as compensation for their time.

### Data Analysis

The focus group discussions were audiotaped, transcribed, and coded by study staff. Standard qualitative methods (ie, data immersion, clustering of preliminary categories, editing, exploring new categories and subcategories, and identifying major themes) were used. Qualitative analysis included (1) individual quotes isolated in transcripts; (2) a hierarchical coding system developed to organize the quotes to capture the full range and depth of participant responses; and (3) the structure, frequency, and interrelationships of the coded quotes to inform interpretation of results. The process included both inductive analysis (moving from facts to theory) and deductive analysis (applying theory to facts). Two independent reviewers reviewed each transcript line-by-line for patterns and themes. ABB developed the initial codebook, which was then further developed and applied by AMB. The team met to resolve coding disagreements, with AES acting as the tiebreaker, although this was not ultimately required.

## Results

### Overview

A total of 8 clinicians participated in the focus groups: 2 psychiatrists (MDs), 1 clinical psychologist (PhD), 1 psychiatric nurse practitioner, 1 licensed mental health clinician, and 3 licensed clinical social workers. All were licensed to practice in Massachusetts; 2 practiced in therapeutic school settings, 1 in a residential setting, 3 in clinic settings, and 2 in private practice. Six were women, 2 were men, 7 self-identified as White and 1 self-identified as a person of color.

### Theme 1: Perspectives

#### Overall Importance of Incorporating Physical Activity and Sleep in Mental Health Treatment

Clinicians were prompted to discuss their perspectives on incorporating physical activity and sleep prescriptions in youth mental health treatment plans. Overall, participants endorsed physical activity and sleep prescriptions as important (8/8, 100%) but not necessarily as a focus of treatment. Clinicians generally recognized a relationship between sleep and physical activity, but they varied in their emphasis on physical activity and sleep during treatment sessions. One clinician who emphasized physical activity and sleep during sessions stated:

I spend quite a bit of time trying to explain to them that the most important piece of their treatment, that we’re all a team...and that physical activity, sleep, and diet would be what I need them to work on first before I can even have an accurate impression of where to go with their treatment or get a baseline of what’s going on with them.

FG2, PT3

Psychiatric diagnosis and prescribed medications were important considerations for clinicians when selecting physical activity or sleep as a target for treatment during sessions with youth:

I tend to talk a little bit more about physical activity just in terms of also medications that the youth may be on and thinking about the impact on physical health and weight...and potentially how that affects sleep and concerns for other medical conditions like sleep apnea.

FG1, PT4

I typically probably ask more about sleep than about physical activity partly because you know they’re frequently coming in with crisis questions.

FG2, PT4

Even with anxiety or things like that, I find that teaching those coping strategies around a sleep routine tends to be more of a first point of intervention. And again, it’s the population, versus depression where I will focus on sleep for sure, but I’m also doing that behavioral activation, and I try to include a lot of physical activity. So, I think it’s contingent on the presenting problem.

FG1, PT2

#### Clinician Confidence in Discussing Health Behaviors During Treatment Sessions With Youth With Psychiatric Diagnoses

Many clinicians, but not all, reported feeling confident and prepared to discuss physical activity (6/8, 75%) and sleep (4/8, 50%) with clients. An emerging theme in this discussion was clinicians’ desire for easy-to-apply clinical guidance. Several clinicians reported having basic training and handouts on these topics but expressed a desire for more guidance on helping youth implement health behavior changes:

I mean as far as maybe education on the importance, fairly comfortable, I could probably use more. But as far as implementing it right, that’s a little more challenging.

FG2, PT3

#### Utility of Physical Activity and Sleep Prescriptions

While most clinicians reported that they did not use physical activity and sleep prescriptions, the majority (6/8, 75%) endorsed a desire to use them and expressed perceived clinical value. However, clarity is still needed for what these interventions should look like based on client age, developmental stage, and other individualized characteristics, as well as on how this guidance can fit within the time limitations of a clinical session:

It’s actually never occurred to me to do sleep prescription or physical activity prescriptions, but I think it’s a fantastic idea.

FG2, PT5

Sleep I feel like I have broad ranges for different age and development...whereas physical activity I’m like, “I don’t know, walk for 10 minutes a day.”

FG1, PT2

I don’t do it. It would be a great idea to do. I think part of where I’ve come from is the time constraints I have kind of don’t allow for some of it, which is unfortunate.

FG2, PT3

#### Use of Screens for Physical Activity

When it comes to engaging in physical activity via screens (ie, apps, exergames, and online coaching), clinicians initially expressed hesitation based on mixed feelings about adolescent screen use. Ultimately, clinicians expressed positive views on using screens to promote physical activity (7/8, 88%), noting their accessibility and acceptability among adolescents:

I’d rather see kids be on an exercise app or something that’s related to their health than social media.

FG1, P1

This speaks to being in step with meeting them where they are. I think that this is, you know, video games and technology. If you speak the language and you can provide this method [of getting active], you’ve got a much better chance of getting them to get actively involved. And so I think that especially for physical activity, this is a great method.

FG2, PT4

### Theme 2: Implementation of GamerFit During Mental Health Treatment

#### Feasibility

Regarding how realistic it is for clients to engage with the GamerFit intervention, clinicians were optimistic that it was doable but noted that it will likely require parental assistance for many youth (eg, reminders and technical support). Clinicians viewed health coaching as a positive aspect of this intervention that might help clients to be successful and noted that other aspects, such as the in-app journal and monitoring system, might be unlikely to be completed on a regular basis. Other feedback included suggestions for potential modifications based on psychiatric diagnoses and emphasis that some features may be especially suited for youth with certain disorders:

The availability of a coach or, or someone that’s meeting with them weekly to, to really kind of work through that would be critical.

FG2, PT6

The barrier to entry might be a little higher for kids with a depressive diagnosis and they might need some extra support in getting started. I also think they would be really well suited to this intervention once they get started.

FG1, PT4

#### Acceptability and Appropriateness

Clinicians believed that the exergaming and coaching components of GamerFit would be most appealing to youth with psychiatric diagnoses. Clinicians indicated that age, gender, and socioeconomic status may impact youth engagement with the intervention. Clinicians also suggested that increased incentives and positive reinforcement, as well as more options for physical activity, would improve engagement. Finally, clinicians believed that the ability to engage with GamerFit at home was a major advantage for those who prefer privacy:

It’s just probably more acceptable than some of the other things that we recommend…the availability of a coach…would be critical.

FG2, PT6

For me it would be kind of age, their diagnosis, whether or not there’s a reward at the end, you know, whether they get any kind of benefit other than just the little trophy at the end.

FG2, PT4

Socioeconomic status...I see kids [without access to school and sport programming] buying in way more versus the kids [with lots of resources].

FG1, PT2

### Theme 3: Approaches for Delivering Prescriptions via GamerFit

#### Incorporation Into Practice

Most clinicians (7/8, 88%) endorsed a willingness to incorporate the GamerFit intervention into their clinical practices. Clinicians expressed enthusiasm about being able to view client engagement with the app to facilitate discussion during sessions, as well as about having resources to share with children and families on the importance of physical activity and sleep, supported by a tangible tool to promote these health behaviors. They were also enthusiastic about the potential for this intervention to be particularly helpful to youth with autism spectrum disorder as they become older and work toward becoming more independent in their health behaviors. An important concern mentioned was a lack of time during outpatient sessions to commit to covering all necessary topics such as health behaviors during regularly scheduled appointments. Other suggestions based on clinician experiences included integrating this intervention into school and residential settings, incorporating a group aspect to promote adherence and engagement, and including additional educational materials to share with clients and families about the role of physical activity and sleep in mental health treatment and how to navigate technology challenges. An additional desire described was advocacy for insurance provisions for coaching and technology coverage to improve family involvement and allow participants to continue the intervention at home after discharge:

I would love to be able to see this in my practice, as well as be able to access the data myself...and be able to talk directly about it...like, “Tuesdays are really hard for you. You don’t get any steps on Tuesdays.” Have those very specific conversations about troubleshooting.

FG1, PT4

You also want to have a fact sheet about the importance of physical exercise and sleep and then be able to step off from that and go, hey, you know...here’s this thing that we are trying and again, can they, will they buy in?

FG2, PT4

[T]he older population of kids on the spectrum, you know we’re looking for independent and we saw obesity and game addiction as critical issues that we were confronting all the time, so I think it’s going to be advantageous.

FG2, PT4

[O]ur kids are in residential and so it seems like an easier intervention to implement in our setting.... I mean really easy, in fact, for us to implement and get almost every kiddo on board, that they would love it, and for us build it right into the rhythm of the day.

FG1, PT1

#### Reporting

Clinicians also provided guidance for preferred methods of accessing, sharing, and using patient data from the GamerFit intervention. Emails integration and incorporation of reporting components into the electronic health record system were identified as the easiest ways to adopt this intervention within clinical practices:

If I were to be able to have [it] sent to me weekly...tracking their progress, how much they’re using it...that would help me kind of see both how effective it is but also kind of what aspects of it are most helpful.

FG2, PT6

One email with all the reports [for clients] instead of individual emails of each participant.

FG1, PT3

## Discussion

Practicing mental health clinicians participated in 1 of 2 focus groups to review the GamerFit intervention and provide feedback on incorporating this program into ongoing mental health treatment to facilitate physical activity and sleep prescriptions. GamerFit was widely perceived as important, clinically feasible, appealing, and appropriate for the target population (ie, youth with common, often comorbid, psychiatric diagnoses). Clinicians identified suggested adaptations to improve clinical workflow and usability as well as suggestions to improve patient engagement.

Many clinicians reported that physical activity and sleep are important aspects of supporting optimal mental health and expressed a desire to incorporate health behavior counseling into their treatment practices. However, they did not feel confident in their training and abilities to incorporate robust eHealth-based physical activity and sleep interventions within sessions with clients. Moreover, there were mixed findings regarding the use of screens for health promotion: some clinicians viewed this as an opportunity to appeal to client interests in media and gaming and to improve accessibility, particularly for those without safe environments for other forms of physical activity, whereas others were more hesitant, given the already high levels of screen time in youth. These findings are consistent with other reports from mental health clinicians on the utility of eHealth in psychiatry, as described by Nicholas et al [31], who found enthusiasm for improving accessibility to treatment in a widely available manner that overcomes resource limitations and simultaneously reduces mental health stigma as a barrier to care. Furthermore, eHealth interventions offer clients the opportunity to access support and to engage with treatment in real time as needed.

In addition to concerns about sedentary behavior associated with screen time, ensuring client confidentiality and data privacy is essential in the design and implementation of eHealth programming. Transparency in data collection purposes and use, as well as established trust between the user, clinician, and the technology or platform itself, are required to overcome this obstacle [31]. eHealth interventions embedded within mental health treatment practices also offer opportunities to enhance real-time self-monitoring, extend clinicians’ assessment of clients within their natural environment (eg, including caregiver involvement), and facilitate data processing to support personalized treatment plans and decision-making [32].

The next finding of this qualitative study was the perceived feasibility and acceptability of the GamerFit intervention in the clinical setting. Overall, the intervention was deemed to be practical, with some aspects perceived to be more engaging than others (eg, health coaching as a critical component, whereas in-app journaling for self-monitoring behavior goals was seen as less likely to be completed). Furthermore, suggestions were made for adapting the intervention based on client age and diagnoses, for example, linking in-app earned rewards with age-appropriate tangible rewards or using the sleep prescription to avoid oversleeping among some youth with depression. The biggest concerns about feasibility and acceptability were potential technical challenges and the likelihood that some clients would require support from parents or caregivers.

Results of this study will be used to help match future features with clinician and client needs (ie, integration into electronic medical records systems and an expanded selection of exergames) [33]. eHealth interventions are widely effective in treating mental health disorders when used alongside psychotherapy, particularly when they are guided (vs unguided) [34], as is the case with the GamerFit intervention. Previous studies have shown that children with psychiatric disorders prefer eHealth interventions that use features such as videos, limited text, personalization options, opportunities to connect with others, and texting reminders [35], all of which are incorporated into the GamerFit intervention. Future iterations of GamerFit will likely provide more evidence on how to best support users with technological challenges, and the inclusion of a health coach is intended to overcome support needs that would typically be required of parents and caregivers. Caregivers are often resource depleted (ie**,** limited in time and energy) and constrained in taking on additional responsibilities. The GamerFit app may help overcome these limitations by applying external accountability and motivation through remote health coaching, thereby encouraging engagement in healthy physical activity and sleep practices.

Limitations of the study included the relatively homogenous sample of participants in the focus groups in terms of race and gender. However, this sample is representative of the demographic majority of the US psychology workforce [36]. An additional limitation is the potential of eHealth interventions to widen the divide in health disparities for those with limited digital literacy and device ownership [31]. Digital literacy and access to technology; data storage; Wi-Fi capabilities; compliance with wearing the Fitbit; and the cognitive abilities and planning skills needed to adhere to the scheduled appointments with the health coach and to complete the physical activity, sleep, and exergaming goals must be considered in the design and implementation of this treatment option.

This study facilitates the translation of a research-based intervention to real-world application for the alleviation of mental and physical health symptoms among a population at elevated risk via direct input from key collaborators (ie, the mental health clinicians). These findings will inform the adaptation and integration of GamerFit as an adjunct to clinical practice with specific considerations for the user (ie, clinician and client) experience, resources, and capabilities.

## Appendix

Multimedia Appendix 1 Semistructured instrument for cross-sectional qualitative data collection across 2 remotely conducted focus groups of 8 mental health clinicians.

Multimedia Appendix 2 Standards for Reporting Qualitative Research reporting checklist.

## Abbreviations

HIPAA: Health Insurance Portability and Accountability Act

## Data Availability

The datasets generated or analyzed during this study are available from the corresponding author on reasonable request.
