# Supplementary material for: Clinician Perspectives on Incorporating Physical Activity and Sleep Prescriptions Using eHealth for Youth With Comorbid Psychiatric Disorders: Qualitative Focus Group Study
Source: JMIR Form Res. 2025 Nov 7;9:e71569. doi: 10.2196/71569 (PMC12639343; doi:10.2196/71569)
Supplement: Multimedia Appendix 1 [file formative_v9i1e71569_app1.docx]

Section A: (20 minutes)

The GamerFit intervention, which we’ve just walked through, is just one approach to improving physical activity and sleep in teens with mental health challenges. We’d like to start by learning more about your perceptions of using physical activity and sleep prescriptions as part of teens’ psychiatric treatment.

1. How important overall do you believe physical activity is to these teens’ mental health treatment?
2. How important overall do you believe sleep is to these teens’ mental health treatment?
3. Which do you think is more important for your clients, adequate physical activity or adequate sleep, and why?
   1. How do you think physical activity and sleep are related?
4. How well prepared do you feel to educate your clients and their families around physical activity literacy?
   1. Sleep literacy?
5. What is your understanding of what a physical activity or sleep prescription is?
6. Do you think physical activity and sleep prescriptions should be a part of clinical treatment? Why or why not?
7. How do you feel about the use of active video games and technology to increase physical activity for teens?

Section B: (20 minutes)

The next questions relate to your perceptions of GamerFit and considerations for implementation in clinical practice.

1. How doable do you think the GamerFit intervention is for the teens you treat?
   1. What parts do you think would be most difficult for teens and families? (technology, adhering to the gaming schedule, watching the videos, putting the tips into practice, meeting with a health coach weekly, using the journal)
   2. What parts do you think are easiest?
   3. How might your perceptions change by diagnosis? Age? Gender?
2. How acceptable do you think the GamerFit intervention is for the teens you treat?
   1. What parts do you believe would be most appealing?
   2. Least appealing?
   3. How might your perceptions change by diagnosis? Age? Gender?
3. How appropriate (suitable, fitting, tailored to) does the GamerFit program seem to be for the teens you treat?
   1. What components would you add to make it more appropriate?
   2. What components would you drop?
   3. How might your perceptions change by diagnosis? Age? Gender?

Section C: (20 minutes)

The last questions relate to your beliefs around if and how GamerFit could be integrated into your clinical practice, treating teens with a variety of mental health challenges.

1. Do you see the GamerFit intervention as something that you could incorporate into your own practice if it existed as a package covered by insurance?
   1. If so, how would you envision doing that?
      1. Are there particular populations you would use it for, and particular populations you would not?
   2. If not, why?
2. If you do see it as something you might consider using, what features would you add to make it easier or more useful in your treatment practice?
   1. What type of report/information would you like to see from this intervention
      1. e.g. a summary of progress/health behavior change, barriers to engagement, attendance to coaching sessions, signs of distress/new or changing psychiatric symptoms, etc.
      2. How would you prefer to receive this information? (e.g. summary in EHR as a visit note, message in EHR from coach)
      3. How often would you prefer to receive this information?
3. If you did integrate it into your practice, how would you approach recommending it to teens and their caregivers?
